# Supplementary material for: Predicting visual function by interpreting a neuronal wiring diagram
Source: Nature. 2024 Oct 2;634(8032):113–23. doi: 10.1038/s41586-024-07953-5 (PMC11446822; doi:10.1038/s41586-024-07953-5)
Supplement: Supplementary file 6 — CRF and ERF predictions for individual TmY4 and TmY9 cells. Analogous to Supplementary Data 3, but for TmY target types. Shown are the top four monosynaptic pathways, the strongest pathway passing through each of the top ten intermediary types (ranking from Extended Data Fig. 7), and the trisynaptic pathway Tm1–TmY–Dm3–TmY (see the section entitled Prediction of spatial normalization). [file 41586_2024_7953_MOESM6_ESM.zip › DataS4/TmY9qΓèÑ/720575940630403948.pdf]

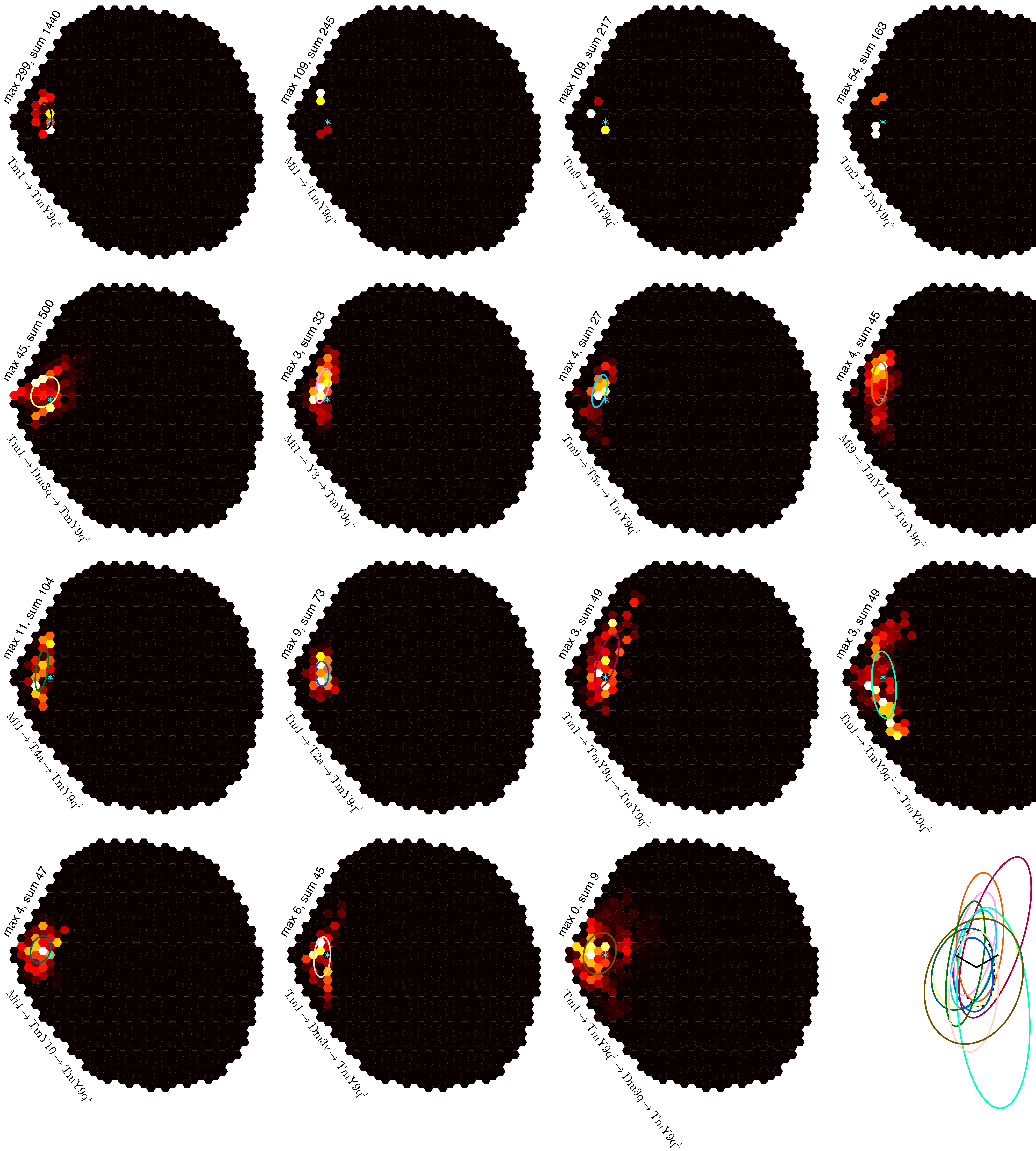

max 299, sum 1440
Tm1 → TmY9q⊥
max 109, sum 245
Mi1 → TmY9q⊥
max 109, sum 217
Tm9 → TmY9q⊥
max 54, sum 163
Tm2 → TmY9q⊥
max 45, sum 500
Tm1 → Dm3q → TmY9q⊥
max 3, sum 33
Mi1 → Y3 → TmY9q⊥
max 4, sum 27
Tm9 → T5a → TmY9q⊥
max 4, sum 45
Mi9 → TmY11 → TmY9q⊥
max 11, sum 104
Mi1 → T4a → TmY9q⊥
max 9, sum 73
Tm1 → T2a → TmY9q⊥
max 3, sum 49
Tm1 → TmY9q → TmY9q⊥
max 3, sum 49
Tm1 → TmY9q⊥ → TmY9q⊥
max 4, sum 47
Mi4 → TmY10 → TmY9q⊥
max 6, sum 45
Tm1 → Dm3v → TmY9q⊥
max 0, sum 9
Tm1 → TmY9q⊥ → Dm3q → TmY9q⊥
